# The effect of a single-strain probiotic administration in the treatment of thermal burns patients

**Published:** 2019-12

**Authors:** David S. Perdanakusuma, Lynda Hariani, Nur Febriany Nasser, Robertus Arian Datusanantyo*

**Affiliations:** Department of Plastic Reconstructive and Aesthetic Surgery, School of Medicine, Universitas Airlangga, Dr. Soetomo General Hospital, Surabaya, Indonesia.

## Abstract

**Background and Objectives:**

Between 2007 and 2011, the mortality rate for burns patients at Dr. Soetomo General Hospital, Surabaya, Indonesia was 14.1% and 60% were suspected to be sepsis-related. Immunosuppression, gut barrier disruption, and intestinal hypomotility cause bacterial and bacterial product translocation. Probiotics improve the intestinal microbiome and eventually reduce bacterial translocation, and an increased secretory immunoglobulin A (SIgA) secretion post-administration of a multi-species probiotic has been observed. We aimed to determine whether a single-strain probiotic administration could show strengthened intestinal immunity, through an increase in SIgA levels, as with multi-strain probiotics.

**Materials and Methods:**

Sixteen burns patients from our hospital Burns Centre were randomized into three treatment groups, and the patients were administered either a placebo, a Lactobacillus reuteri protectis probiotic, or a Bifidobacterium infantis 35624 probiotic for 14 consecutive days. The SIgA levels were analyzed using ELISA pre- and post-treatment.

**Results:**

The post-treatment SIgAlevelsin the placebo, Lactobacillusreuteri protectis probiotic, and Bifidobacterium infantis 35624 probiotic groups were 222.56±74.22 mg/dL, 223.92±68.89 mg/dL, and 332.38±64.27 mg/dL, respectively. Decreased SIgA levels were observed in the placebo (7.19±15.87) and in the Lactobacillus reuteri protectis probiotic (1.9920±14.76) groups, whereas an increase was seen in the SIgA level in the Bifidobacterium infantis 35624 probiotic group (58.26±77.41).

**Conclusion:**

The Bifidobacterium infantis 35624 single-strain probiotic is generally superior to Lactobacillus reuteri protectis in altering intestinal immunity; however, this finding was not statistically significant. A multi-strain probiotic supplement is recommended for burns patients.

